# The effect of genotype and in utero environment on interindividual variation in neonate DNA methylomes

**DOI:** 10.1101/gr.171439.113

**Published:** 2014-07

**Authors:** Ai Ling Teh, Hong Pan, Li Chen, Mei-Lyn Ong, Shaillay Dogra, Johnny Wong, Julia L. MacIsaac, Sarah M. Mah, Lisa M. McEwen, Seang-Mei Saw, Keith M. Godfrey, Yap-Seng Chong, Kenneth Kwek, Chee-Keong Kwoh, Shu-E. Soh, Mary F.F. Chong, Sheila Barton, Neerja Karnani, Clara Y. Cheong, Jan Paul Buschdorf, Walter Stünkel, Michael S. Kobor, Michael J. Meaney, Peter D. Gluckman, Joanna D. Holbrook

**Affiliations:** 1 Singapore Institute of Clinical Sciences (SICS), A*STAR, Brenner Centre for Molecular Medicine, Singapore 117609;; 2 School of Computer Engineering, Nanyang Technological University (NTU), Singapore 639798;; 3 Centre for Molecular Medicine and Therapeutics, Child and Family Research Institute, Department of Medical Genetics, University of British Columbia, Vancouver, BC V5Z 4H4 Canada;; 4 Saw Swee Hock School of Public Health, NUS, Singapore 117597;; 5 MRC Lifecourse Epidemiology Unit and NIHR Southampton Biomedical Research Centre, University of Southampton and University Hospital Southampton NHS Foundation Trust, Southampton, SO16 6YD, United Kingdom;; 6 Yong Loo Lin School of Medicine, National University of Singapore, National University Health System, Singapore 119228;; 7 KK Women’s and Children’s Hospital, Singapore 229899;; 8 Ludmer Centre for Neuroinformatics and Mental Health, Douglas University Mental Health Institute, McGill University, Montreal, (Quebec) H4H 1R3 Canada;; 9 Centre for Human Evolution, Adaptation and Disease, Liggins Institute, University of Auckland, Auckland 1142, New Zealand

## Abstract

Integrating the genotype with epigenetic marks holds the promise of better understanding the biology that underlies the complex interactions of inherited and environmental components that define the developmental origins of a range of disorders. The quality of the in utero environment significantly influences health over the lifecourse. Epigenetics, and in particular DNA methylation marks, have been postulated as a mechanism for the enduring effects of the prenatal environment. Accordingly, neonate methylomes contain molecular memory of the individual in utero experience. However, interindividual variation in methylation can also be a consequence of DNA sequence polymorphisms that result in methylation quantitative trait loci (methQTLs) and, potentially, the interaction between fixed genetic variation and environmental influences. We surveyed the genotypes and DNA methylomes of 237 neonates and found 1423 punctuate regions of the methylome that were highly variable across individuals, termed variably methylated regions (VMRs), against a backdrop of homogeneity. MethQTLs were readily detected in neonatal methylomes, and genotype alone best explained ∼25% of the VMRs. We found that the best explanation for 75% of VMRs was the interaction of genotype with different in utero environments, including maternal smoking, maternal depression, maternal BMI, infant birth weight, gestational age, and birth order. Our study sheds new light on the complex relationship between biological inheritance as represented by genotype and individual prenatal experience and suggests the importance of considering both fixed genetic variation and environmental factors in interpreting epigenetic variation.

The relationship between interindividual variation in the epigenome—especially DNA methylation—and disease risk is an area of intense research interest. Although the effect of fixed genetic variation on DNA methylation is apparent in studies of allele-specific methylation and genomic imprinting, there is also emerging evidence for environmental influences as a source of epigenomic variation. Perinatal cohort studies offer a unique opportunity to explore the origins of variation across the epigenome, and in particular the extent to which fixed genetic variation can moderate the relationship between prenatal environmental factors and epigenetic status at birth.

Epidemiological data link disease risk directly to the in utero environment (Roseboom et al. 2001; Gillman et al. 2003; Hillier et al. 2007; Painter et al. 2008; Alisi et al. 2011; Sohi et al. 2011; Dancause et al. 2012; Schwarze et al. 2012) or to birth outcomes as a surrogate for the in utero environment (Barker et al. 1989; Hofman et al. 2004; Boney et al. 2005; Bouhours-Nouet et al. 2008; Broekman et al. 2009; Skilton et al. 2011). This phenomenon is often called fetal programming and defines, in part, the developmental origins of health and disease (Bjornsson et al. 2004; Gluckman et al. 2008).

Stable alterations to the epigenome are considered to be a putative molecular mechanism for fetal programming. Thus the environmental epigenetic hypothesis (Seckl and Meaney 1993; Meaney and Ferguson-Smith 2010) suggests that the in utero environment affects the epigenome and that resulting epigenetic marks alter physiology to affect later disease risk (Gluckman et al. 2009). Evidence for this hypothesis derives from studies documenting the relationship between specific DNA methylation marks, in utero environments, and later phenotypes (Guo et al. 2008; Perera et al. 2009; Pilsner et al. 2009, 2012; van der Kaay et al. 2009; Feinberg et al. 2010; Kaminen-Ahola et al. 2010; Ollikainen et al. 2010; Fryer et al. 2011; Hoyo et al. 2011; Tobi et al. 2011; Relton et al. 2012; Stunkel et al. 2012). For example, maternal carbohydrate intake in early pregnancy and offspring adiposity at 9 yr of age associate with the DNA methylation level of the *RXRA* promoter in the umbilical cord (Godfrey et al. 2011). There are examples of persistent, environmentally induced DNA methylation states. For instance, variations in maternal care alter the methylation of the glucocorticoid receptor gene promoter in rats (Weaver et al. 2004), and differential methylation at this same region is associated with childhood trauma in humans (McGowan et al. 2009). Although many aspects of the epigenome such as histone modification are likely to be involved, current evidence concentrates mostly on DNA methylation.

Multiple epigenome-wide association studies (EWAS) within birth cohorts have been initiated to search for epigenomic signatures of early life environment that may influence later life phenotype (Ng et al. 2012; Michels et al. 2013; Mill and Heijmans 2013). However, at least some DNA methylation marks are specified by sequence context in *cis* (Brandeis et al. 1994; Lienert et al. 2011), precluding exclusive environmental regulation. In humans, interindividual variation in DNA methylation could be wholly or in part a consequence of nucleotide polymorphism, as local genetic variants influence the propensity for methylation of neighboring cytosines. These polymorphisms can be defined as methylation quantitative trait loci (methQTLs). MethQTLs have been postulated as a link between GWAS SNPs and phenotype (Liu et al. 2013).

Gibbs et al. (2010) showed methQTLs in human tissue in studies of multiple human brain regions and found between 4% and 5% of the 27,000 CpG sites studied had methylation levels that were significantly dependent on genotype (as measured in 537,411 SNPs). Although both *cis* (defined as the CpG and SNP pair being within 1 Mb of the chromosomal region) and *trans* effects were detected in this study, *cis* methQTLs were in massive abundance. The average distance between a SNP and CpG pair that made up a significant methQTL was 81 kb, but the peak enrichment across the *cis* methQTLs was 45 bp. Zhang et al. (2010) examined human cerebellum and found 9% of CpGs tested (748/8590) were within methQTLs. Another study (Bell et al. 2011) detected 180 (1%) methQTLs in the 22,290 CpGs and 3 million SNPs investigated across 77 HapMap lymphoblastoid cell lines, five of which were also reported by Gibbs et al. (2010). Most methQTLs were found in *cis* and over short distances of <5 kb. Moreover, genotype at one SNP associated with methylation at multiple neighboring CpGs, as might be expected given the positional correlation previously noted in methylation data (Eckhardt et al. 2006). Similar results were obtained by Grundberg et al. (2013), who also discovered methQTLs in adult adipose tissue acting in *cis*, which explains 19% of the observed variance in methylation levels.

Ethnicity can be used as a proxy for genotype and has been shown to influence the DNA methylome (Zhang et al. 2011). African and European individuals have population-specific patterns of DNA methylation at ∼30% of CpGs measured. Methylation levels at ∼50% of these population-specific CpGs are explained by divergence in allele frequencies at *cis*-acting SNPs between populations (Fraser et al. 2012). Studies of methylation differences using Illumina InfiniumHumanMethylation450 BeadChip array data from 133 lymphoblastoid cell lines from European and African HapMap samples found that 13% of analyzed CpGs showed significant methylation differences between the populations, >50% of which were in methQTLs with local SNPs (Moen et al. 2013). CpGs showing differential methylation levels across ethnicities are more likely to be driven by genotype than other CpGs (Heyn et al. 2013). Nevertheless, these studies also found that some methQTLs are specific to one population with no correlation between genotype and methylation in other populations, suggesting possible gene × environment interactions.

Methylomes are more similar in related than unrelated individuals (Bjornsson et al. 2008), and concordance tracks degree of relatedness; the methylomes of monozygotic twins are more closely related than those of dizygotic twins (Kaminsky et al. 2009). Methylation profiles from three different tissues of twin neonates generated on the InfiniumHumanMethylation27K BeadChip only clustered into twin pairs between 29% and 71% during unsupervised analysis, suggesting nongenomic influences on the newborn methylome (Gordon et al. 2012).

In this article, we focus on the examination of the relative influences of genotypic, environmental, and gene × environment interactive effects on the neonatal methylome. Recent studies describe evidence for gene × environment interactions (G × E effects) on DNA methylation. Yousefi et al. (2013) found that the *LEPR* genotype interacted with maternal smoking to associate with methylation of *LEPR*. A SNP within the *IL4R* gene combined with methylation at a CpG site within the same gene predicts the risk of childhood asthma (Soto-Ramirez et al. 2013). Moreover, Klengel et al. (2013) found that interaction of the *FKBP5* genotype and early childhood trauma affects methylation of *FKBP5* intron 7, *FKBP5* expression, and subsequent deregulation of glucocorticoid receptor signaling. The proportions of interindividual variation in methylomes that are driven by genotype, environment, or an interaction of gene and environment (G × E) are currently unknown. To clarify the relative influence of gene and in utero environment on epigenetic status at birth, we studied the variation in genome-wide DNA methylation patterns in umbilical cord samples from 237 Asian neonates using the InfiniumHumanMethylation450 BeadChip together with genotyping and extensive measures of in utero environmental conditions. We report that genotype, and in particular G × E interactions, explain substantial proportions of interindividual variation in the methylome at birth.

## Results

### Ethnicity associates with the first components of genotype but not DNA methylation

**Figure 1.**
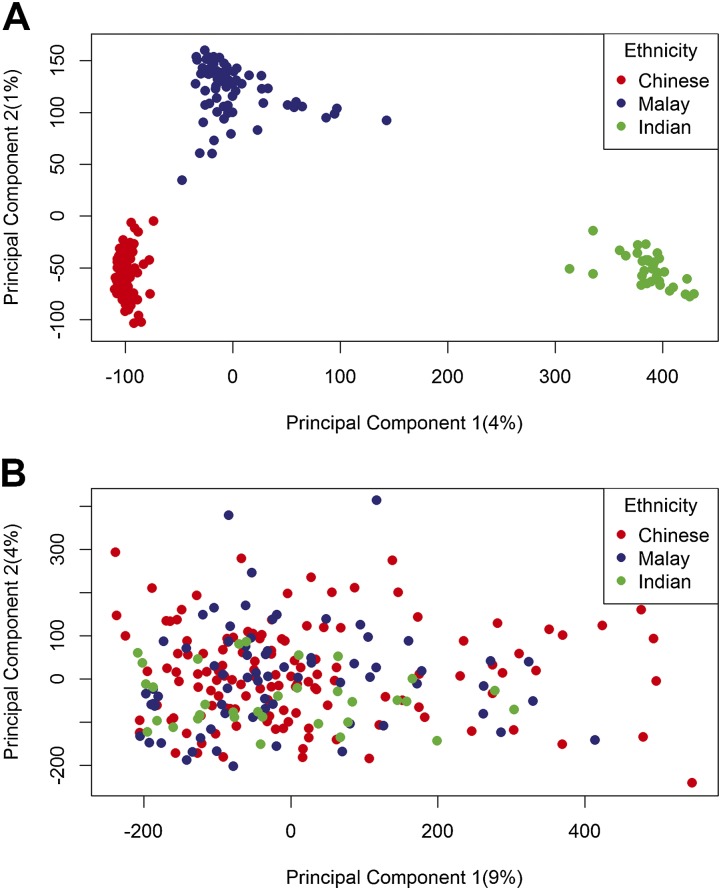
(*A*) Unfiltered genotypes organize subjects by ethnicity. Principal component 1 (*x*-axis) plotted against principal component 2 (*y*-axis) from principal component analysis of genotypes for all 708,365 heterologous SNPs across 237 subjects. Subjects are colored by self-reported ethnicity. (*B*) Unfiltered methylomes do not organize subjects by ethnicity. Principal component 1 (*x*-axis) plotted against principal component 2 (*y*-axis) for principal component analysis of methylation levels at all 301,468 variable CpGs across 237 subjects. Subjects are colored by self-reported ethnicity.

Umbilical cord tissue DNA from 237 individuals (131 Chinese, 72 Malay, 34 Indian) in the GUSTO birth cohort (Soh et al. 2013) were interrogated on both Illumina OmniExpress + Exome genotyping arrays and InfiniumHumanMethylation450 BeadChip; 708,365 SNPs (from the 958,178 assayed) varied in genotype and 301,468 CpGs (from the 411,107 assayed) varied in methylation levels by >5% across the 237 individuals under study (for study subject characteristics, see Table 1). When the genotype data were subjected to principal component analysis, the samples were cleanly separated by ethnicity. The Indian subjects clustered away from Chinese and Malay subjects on principal component 1, and the Chinese and Malay subjects separated on principal component 2 (Fig. 1A). In contrast, when the DNA methylation data were subjected to principal component analysis, the samples did not separate well by ethnicity on components 1 or 2 (Fig. 1B). The methylation and genotyping arrays do measure different subsets of the genome; however, each have probes in every coding gene in the genome and so are broadly comparable. If methylation levels were specified wholly by genotype, we would expect ethnicity to drive the methylome in the same manner as observed for the genotype.

**Table 1.**
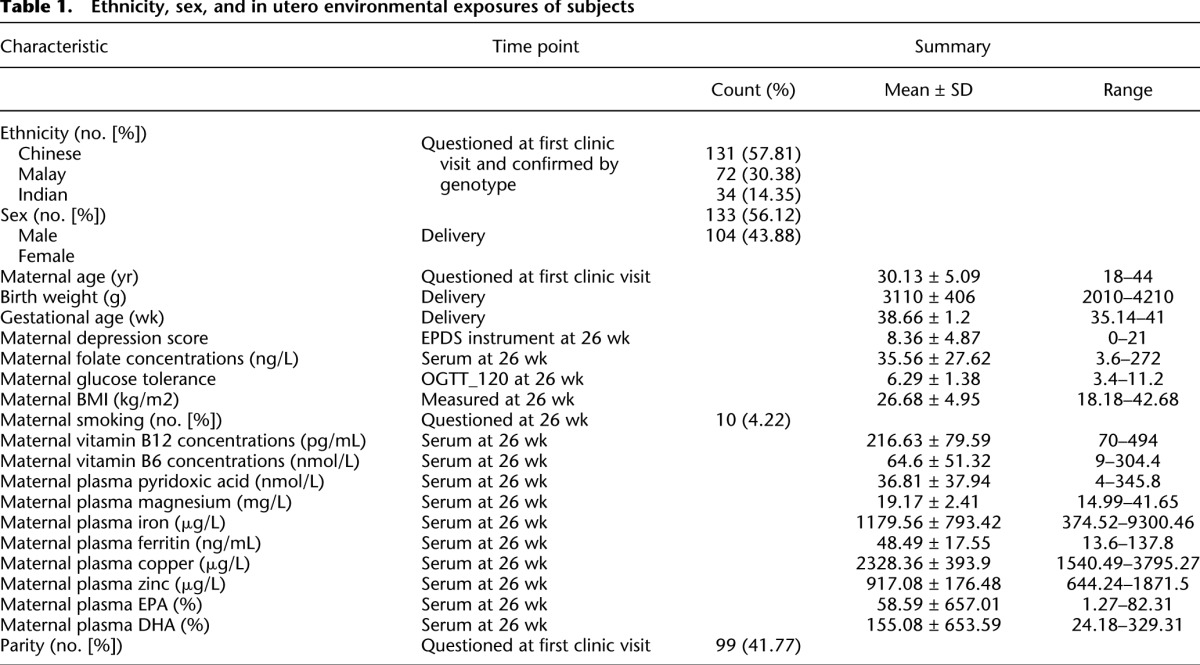
Ethnicity, sex, and in utero environmental exposures of subjects

### Evidence for methQTLs

Methylation levels at the majority of CpGs in the neonate methylomes were very similar across individuals, with median absolute deviation scores (MAD) <0.1. To identify regions with appreciable interindividual variance that could reflect individual in utero experience or genotype, we defined interindividual variably methylated regions (VMRs) across the 237 individuals using a previously published methodology (Ong and Holbrook 2014). This analysis returned 1423 VMRs; these were found on every chromosome in a manner roughly dependent on probe spacing (Supplemental Table 1; Supplemental Fig. 1), although they tended toward north shore (*P* = 1.77 × 10^−4^) and open sea (*P* = 9.11 × 10^−12^) regions (Supplemental Table 2).

The CpG with the highest MAD score within each VMR was used to represent each of the 1423 VMRs and denoted as VMR-CpGs. For each VMR-CpG, we compared methylation levels to SNP genotype at all 708,365 heterologous positions by linear regression, controlling for sex. Strong sex effects on the autosomes have previously been noted in InfiniumHumanMethylation450 BeadChip data and are at least partially artifactual, driven by cross-reaction of probes with the sex chromosomes (Chen et al. 2013).

**Figure 2.**
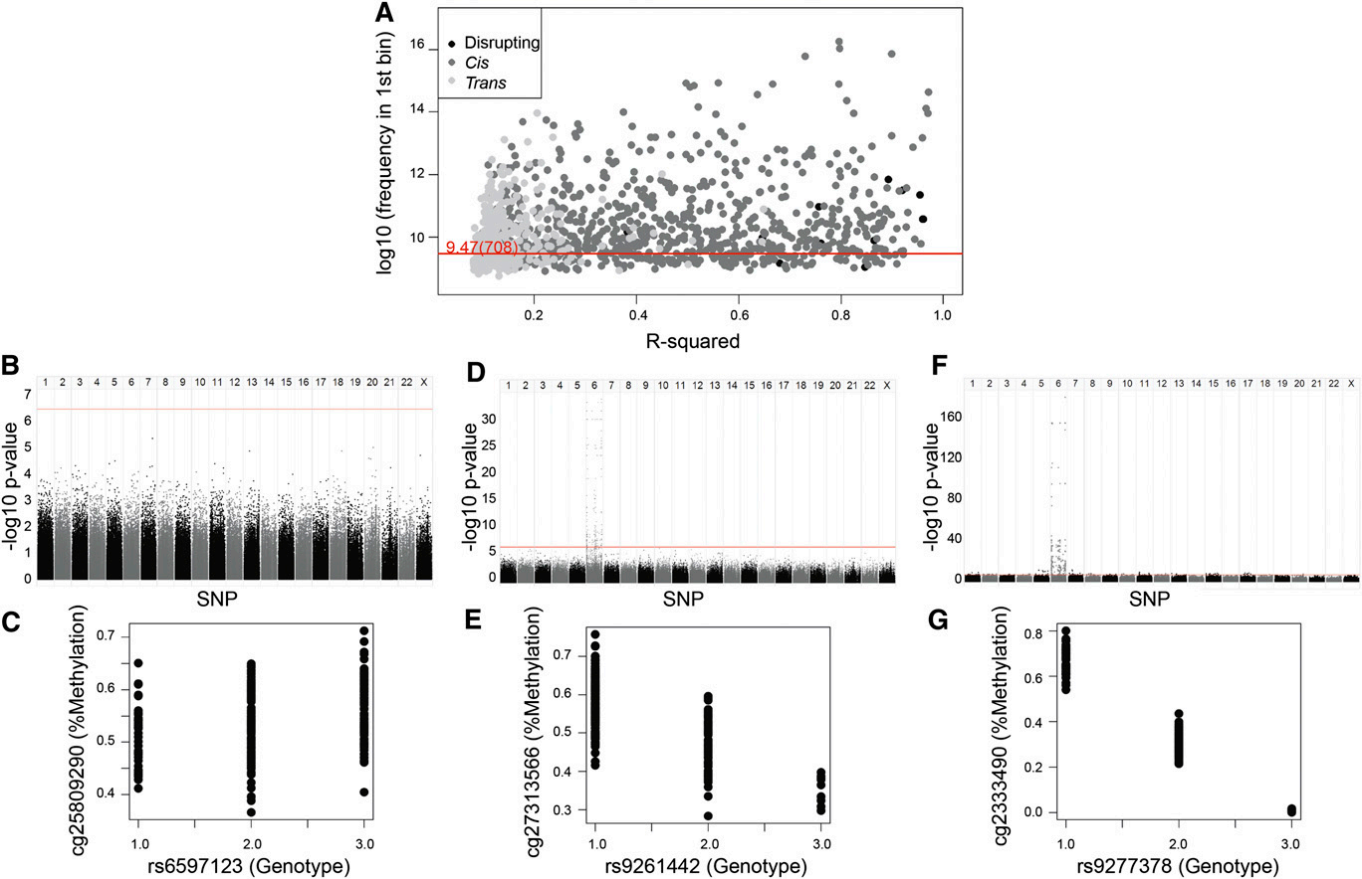
(*A*) The strength of association between genotype and methylation levels is a continuum, with most VMR-CpGs showing some association with genotype. Scatter plot of Pearson R^2^ (*x*-axis) for the VMR-CpG and best SNP match against log_2_ of the number of regression *P*-values in the first bin of 1000 equally distributed bins (*y*-axis); the red line represents an absolutely even 708 regression *P*-value in the first bin. (Black) Disrupting pairs, (dark gray) *cis* pairs, (light gray) *trans* pairs. (*B*) Some VMR-CpGs are minimally influenced by genotype. Manhattan plot of methylation at one VMR-CpG against all SNPs (*x*-axis) with −log_10_ *P*-value on the *y*-axis, as an example of VMR-CpG with low R^2^ and a low number of *P*-values in the first bin. (*C*) Scatter plot of genotype (*x*-axis) against methylation (*y*-axis) for the top pair from the same VMR-CpGs as in *B*. (*D*) Some VMR-CpGs are moderately influenced by genotype. Manhattan plot of methylation at one VMR-CpG against all SNPs (*x*-axis) with −log_10_ *P*-value on the *y*-axis, as an example of VMR-CpG with moderate R^2^ and a moderate number in the first bin. (*E*) Scatter plot of genotype (*x*-axis) against methylation (*y*-axis) for the top pair from the same VMR-CpGs as in *D*. (*F*) Manhattan plot of CpG against all SNPs (*x*-axis) with −log_10_ of the *P*-value (*y*-axis), as an example of VMR-CpG with high R^2^ and a high number in the first bin. (*G*) Scatter plot of genotype (*x*-axis) against methylation (*y*-axis) for the top pair from the same CpG as in *F*.

The best-match (lowest *P*-value) SNP for each VMR-CpG was retained. The final data set of 1423 CpG–SNP pairs showed a range of associative *P*-values skewed toward the low end, suggestive of methQTLs in the data set (Supplemental Fig. 2). *P*-values obtained from the regression analysis were binned into 1000 equally spaced bins. Distributions were defined as skewed if the first bin contained more than the 708 *P*-values expected for each bin if *P*-values were distributed evenly. Nine hundred sixty-six (68%) of the individual VMR-CpGs had a skewed *P*-value distribution. In addition, 1037 (73%) had a –log_10_ *P*-value above 6.5, which is approximately the background noise level seen across the genome (Fig. 2B,D,F).

### Best-matched SNP and VMR-CpG pairs tended to be closely colocated in *cis*

Twelve of the 1423 CpG–SNP pairs (<1%) included a SNP located within the CpG that either creates or eliminates a CpG site. We denoted these instances as “disrupting pairs.” Eight hundred twenty-eight of the 1423 pairs (58%) included a SNP and CpG from the same chromosome. We denoted these as “*cis* pairs.” The remaining 583 pairs (41%) include a CpG and SNP located on different chromosomes and were denoted as “*trans* pairs.” The proportion of *cis* pairs was much greater than expected by chance (chance would predict pairs to be equally distributed across chromosomes at ∼4.5%) and tended to be more likely to come from a skewed *P*-value distribution and to have higher R^2^ values than the *trans* pairs (Fig. 2A). The influence of a genotype operating independently of environmental context was undetectable for some VMR-CpGs (e.g., Fig. 2B,C) and very clear for others (e.g., Fig. 2F,G). Even in a mid-range where methylation at the VMR-CpG was not strongly related to genotype on a scatter plot (e.g., Fig. 2E), there was still a tendency for the best hit to be in *cis* (e.g., Fig. 2D).

**Figure 3.**
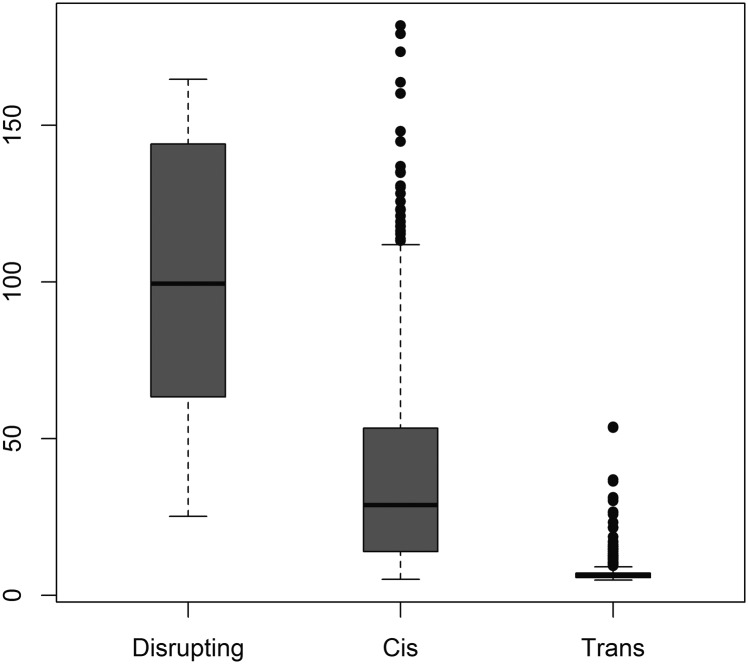
The strength of association between genotype and methylation is strongest for disrupting pairs and weakest for *trans* pairs. Box plot of –log_10_ of the *P*-value of the association between genotype and methylation levels at each VMR-CpG, for CpG–SNP pair categories disrupting (SNP is within CpG), *cis* (SNP is on same chromosome as CpG), and *trans* (SNP is on a different chromosome to the CpG).

**Figure 4.**
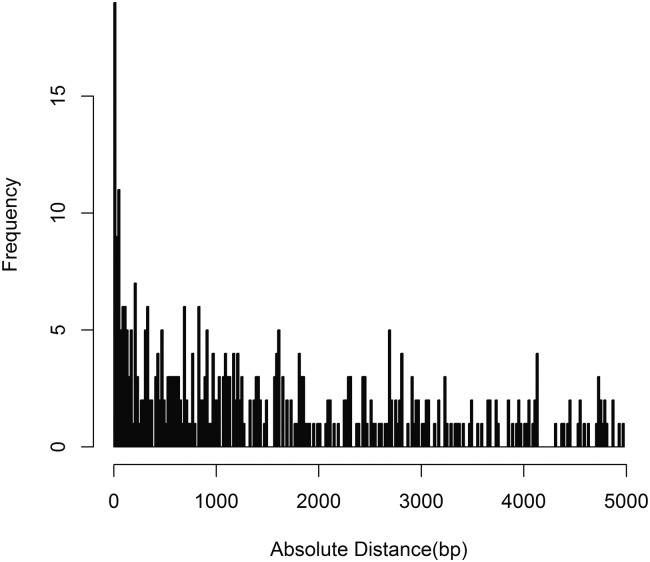
*Cis* pairs tend toward short distances between the SNP and CpG. Bar chart of –log_10_ of the *P*-value (*y*-axis) against the chromosomal distance between the SNP and CpG (*x*-axis) for c*is* pairs within 5 kb.

Disrupting pairs had significantly stronger associations between genotype and methylation level than *cis* pairs, which in turn had stronger associations than *trans* pairs (*P* = 7.3 × 10^−117^ by Kruskal-Wallis test) (Fig. 3). Within the *cis* pairs, there was a strong inverse relationship for the strength of association between methylation levels and genotype, and the distance between the CpG and SNP (*P* = 8.84 × 10^−5^). The mode was within 0–10 bp or 50–60 bp without the disrupting pairs (Fig. 4). There seemed to be a continuum of genotypic influence on methylation levels, with ∼68%–73% of the VMR-CpGs showing an appreciable association with genotype. Defining a genuine methQTL would necessitate choosing arbitrary cut-offs. However, the *trans* CpG–SNP pairs tended to be on the lowest end of the distribution of association statistics and hence were less likely to represent genuine methQTLs (Fig. 2A).

### G × E models best explained variation in methylation at most VMR-CpGs

**Figure 5.**
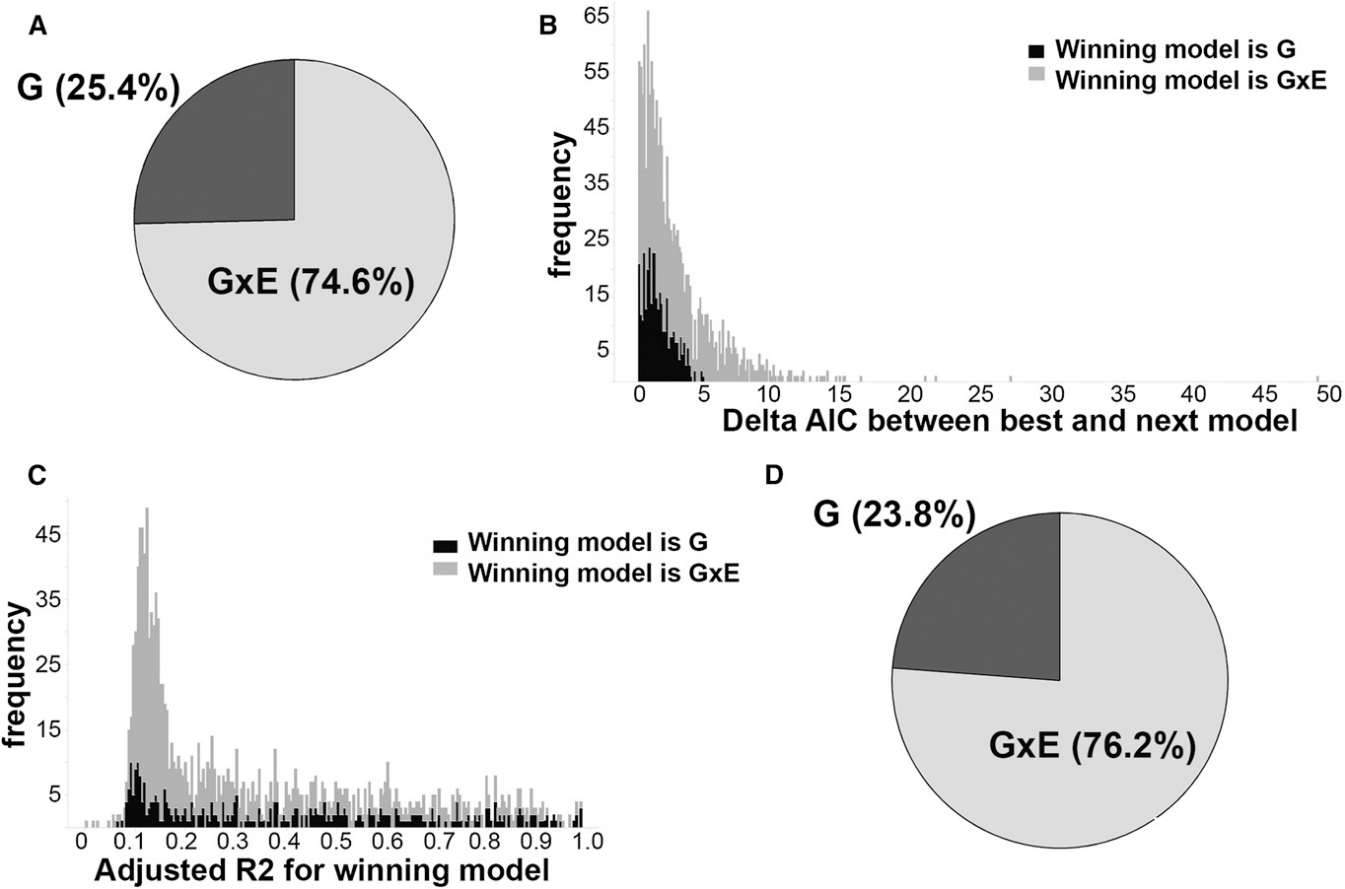
(*A*) The majority of VMR-CpGs are best explained by G × E models. Pie chart showing the proportions of 1423 VMR-CpGs, which are best explained by the genotype (G), environment, or interaction between gene and environment (G × E) regression models. (*B*) Genotype tends to be a narrow winner. Stacked histogram of deltas between delta AICs for best and next-best model across 1423 VMR-CpGs. Each box is colored to denote the model that best explained methylation levels at the VMR-CpG. (*C*) The models explain the range of variation at VMR-CpGs. Stacked histograms of adjusted R^2^ of the winning model across all 1423 VMR-CpGs. Each box is colored by the winning model. (*D*) The proportion of VMR-CpGs explained by G × E is stable as model confidence increases. Pie chart showing the proportions of 210 VMR-CpGs that were best explained by the genotype, environment, or G × E regression models with no substantial support for the next-best model (Δ > 2) and adjusted R^2^ > 0.4.

The GUSTO study has ascertained multiple environmental measures that are known to influence or reflect the quality of the in utero experience. We chose 19 parameters as surrogate measures of the uterine environment (see Table 1) and examined whether methylation at each of our 1423 VMR-CpGs was better explained by genotype, environment, or G × E. We ran 39 regression models (see Methods), including the best SNP hit from the previous methQTL analysis, described above. For the G × E models, we tested all the SNPs located on the same chromosome as the CpG, as well as the best hit from the methQTL analysis. All models contained sex and were compared using Akiake information coefficients (AICs). Methylation levels at ∼25% of the 1423 VMR-CpGs were best explained by genotype alone, while the rest were best explained by G × E models (Fig. 5A). The models containing environment alone were never the best explanation of methylation at the VMR-CpGs. The information loss experienced between the two top models (Δ) (Burnham and Anderson 2004) ranged from 0.0002–49.0 (calculated by delta in AICs). The genotype-only model tended to be a “narrow winner”; i.e., the Δ was low in comparison to the Δ shown by the cases where G × E was the best explanation (Fig. 5B). The majority of best models showed an adjusted R^2^ value above 0.12. The distribution of adjusted R^2^ values for the cases where G × E was the best model was shifted slightly to the right compared with that for the cases of genotype being the best model (Fig. 5C). When the results were restricted to models without substantial support for the next best model, with Δ > 2 (Fig. 5D), and an adjusted R^2^ > 0.4, the proportion of VMR-CpGs for which genotype only was the best model was still ∼24%.

The VMR-CpGs that were best explained by genotype alone tended to be in open seas (*P* = 1.38 × 10^−5^), while VMR-CpGs that were best explained by G × E tended to be both in open seas (*P* = 2.12 × 10^−6^) and north shores of CpG islands (*P* = 0.012) (Supplemental Table 2; Supplemental Fig. 1), consistent with previous observations (Feinberg et al. 2010). Once again the majority of the SNPs in the best models were in *cis* with the VMR-CpGs (85% for the winning G × E models and 78% for the G winning models). When only models with high levels of support (Δ > 2 and adjusted R^2^ > 0.4) are included, only *cis* SNPs remain (Supplemental Fig. 3).

**Figure 6.**
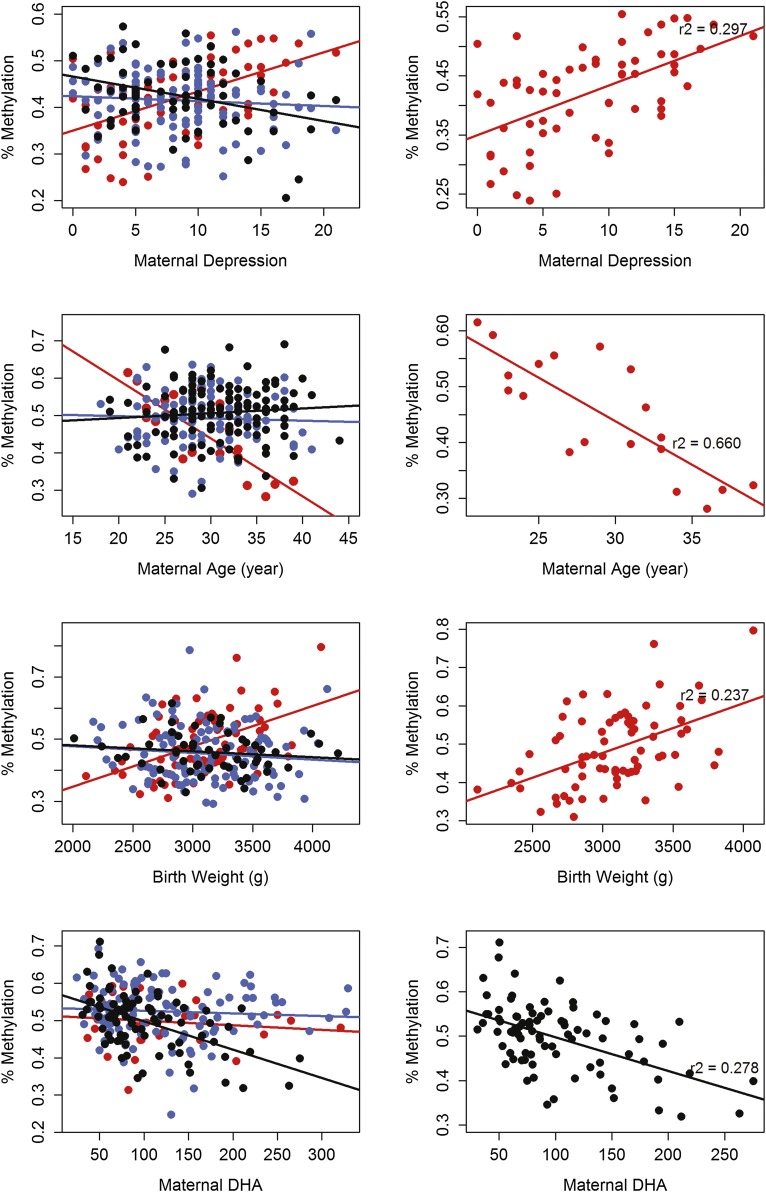
Association of methylation with environment in one genotypic group. Examples of VMR-CpGs whose methylation levels are significantly associated with phenotype in only one genotypic group. Phenotypic values are shown on the *x*-axis, and methylation value in percentages on the *y*-axis. (*Left*) Data for all samples. Samples are colored by their genotypic group (red indicates AA; blue, AB; black, BB), and a straight fit line is fitted for each group. (*Right*) Genotypic subgroup with the highest R^2^.

The G × E models allow for methylation associated with environment in each of the genotypic subgroups present but with different slopes. However, we often saw that environment and methylation were very closely associated in one genotype and less so in the other two genotypes. To identify these CpG-VMRs, we segregated the subjects by the genotype in the best G × E model and ran regressions of methylation and the phenotype for the best G × E model. Fifty VMR-CpGs were significant after Bonferroni correction. Examples are shown in Figure 6.

### Restricting subjects to one ethnicity reduced the impact of genotype

The 491 VMR-CpGs with differential methylation for ethnicity have significantly stronger associations with SNPs compared with all other 930 CpGs (*P* = 2.17 × 10^−19^). We reasoned that the inclusion of multiple ethnicities would increase the genotypic influence on the methylome. We thus conducted an analysis including only the 131 Chinese subjects in our data set. In the Chinese-only subgroup, we still found many methQTLs, but there was a subtle (downward) shift to less significant associations for the majority of CpG–SNP pairs (Supplemental Fig. 4A). The number of VMR-CpGs for which the methylation was best explained by genotype alone decreased slightly to 21% (Supplemental Fig. 4B).

### CpGs with the most variation across samples are most likely to be driven by genotype

**Figure 7.**
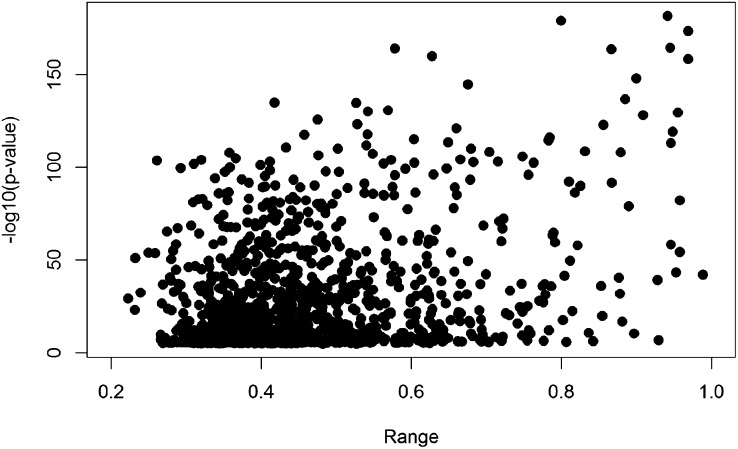
VMR-CpGs with larger ranges of methylation values are more likely to be MethQTLs. Scatter plot showing the range of methylation values at each CpG across samples (*x*-axis) compared with the strength of association between the VMR-CpG methylation values and the genotype of the best SNP (*y*-axis).

We noted that the CpG MAD score across samples or the range of methylation values across samples was related to the strength of association between the CpG methylation values and the genotype of the best SNP (*P* = 3.54 × 10^−39^) (Fig. 7). Our 1423 VMR-CpGs were chosen to lie within VMRs. This approach improves the specificity of the analysis (Ong and Holbrook 2014). However, we also picked a further 1500 CpGs that had the most extreme MAD scores in the data set and that were not included in a VMR. Indeed, within this set the genotype-driven model is a better explanation for a higher proportion of the CpGs (Supplemental Fig. 5).

## Discussion

We used DNA obtained from the umbilical cord at birth to provide evidence for the influence of genotype on interindividual variability in DNA methylation. MethQTLs were readily apparent in the methylome of neonates born at term with birth weights that spanned the normal range from a community sample of pregnancies that were not selected or weighted for any particular outcome. Our findings are consistent with a recent report (Gutierrez-Arcelus et al. 2013) of 14,189–32,318 methQTLs (3%–7%) found within Infinium450K data by searching the SNPs (genotype 2.5 million) in 5-kb flanking regions on either side of each CpG in umbilical cord tissue and cord blood. However, Gutierrez-Arcelus et al. (2013) did not describe any phenotypic or environmental data on their subjects or attempt to show any environmental influence on methylation levels. In these ways, our studies differ in aims and scope. We found that the effect of genotype on methylation was more pronounced in multiethnic populations but was also apparent within the sample of homogeneous ethnicity (Fig. 5; Supplemental Fig. 4B). Moreover, there was a strong relationship between the range of methylation values and influence of genotype (Fig. 7; Supplemental Fig. 5). There is recent evidence that epigenetic states may serve to directly mediate the relation between certain genetic polymorphisms and phenotype (McVicker et al. 2013). The mechanism by which sequence polymorphism affects local CpG methylation is unknown (assuming the SNP is not within the CpG, a situation we have labeled disrupting pairs). However, the effect is likely mediated by the sequence-specific binding of proteins and alteration of chromatin factors that subsequently affects binding of de novo methylases or demethylating mechanisms. A similar mechanism has been evidenced for other epigenetic marks, recently shown to be affected by polymorphism in a similar way to methylation (Otani et al. 2009; Kasowski et al. 2013; Kilpinen et al. 2013; McVicker et al. 2013). A study of these other epigenomic marks in a similar context to that we describe for DNA methylation would be fascinating.

Despite the evidence for genotypic influence independent of environmental influences, we found that the majority of the VMR-CpGs were best explained by the interaction of genotype and maternal/fetal environment (Fig. 5). The environmental measures selected for analysis included proxy factors such as birth weight and gestational age, maternal smoking and depression, all of which are known to influence a broad range of developmental outcomes. Despite the importance of G × E models in explaining the majority of VMR-CpGs, we failed to find VMR-CpGs that were best explained by environmental conditions independently of genotype. This finding emerged despite the considerable evidence for the effects of environmental factors in pregnancy on both epigenetic states (Barker et al. 1989; Roseboom et al. 2001; Gillman et al. 2003; Hofman et al. 2004; Boney et al. 2005; Hillier et al. 2007; Bouhours-Nouet et al. 2008; Painter et al. 2008; Broekman et al. 2009; Alisi et al. 2011; Skilton et al. 2011; Sohi et al. 2011; Dancause et al. 2012; Schwarze et al. 2012) and health outcomes (Godfrey et al. 2011). Our data suggest that genotype exerts a moderating influence on such environmental effects.

The regression models explained a high proportion of the variance in methylation levels for a minority of the VMR-CpGs. For most VMR-CpGs, the models explained only 10%–20% of the variance (Fig. 5D). One reason why the models might fail to explain the majority of the variance for more VMR-CpGs is that the association between methylation and a specific environmental condition may only be apparent in one genotypic subgroup (Fig. 6). This observation is consistent with the emerging view that genotype can determine the degree of environmentally induced phenotypic plasticity (i.e., so-called “plasticity genes”) (Belsky et al. 2009; Simons et al. 2011) and that epigenetic mechanisms serve to maintain environmentally induced phenotypic variation (Meaney and Ferguson-Smith 2010; Pujadas and Feinberg 2012). This is a particularly important finding for studies of either population differences or the influence of environmental factors on phenotypic outcomes. The finding of G × E interaction effects on the epigenome suggests that the necessary level of interrogation extends beyond simple EWAS analysis to include genotype. The failure to include assessment of genotypic moderation of environment–epigenome relations might result in an underestimation of the potential for environmental impact among subpopulations. Patel et al. (2013) show that candidate SNP and CpG loci with marginal associations in GWAS and EWAS, respectively, can show strong associations with disease (in this case type 2 diabetes) when combined.

Another possible reason for the modest explanatory power of certain models is that they are limited to only 19 proxy factors related to the in utero environment and that only single individual genotypic and environmental influences were examined within the models. Models containing multiple SNPs (epistasis) or combining different facets of environments may explain more of the variance in DNA methylation. Moreover, the population under study did not show extreme values in the environmental measures (Table 1), and stronger effects may emerge with the study of high-risk populations.

Cellular heterogeneity may also compromise our ability to account for variance across the methylome. The umbilical cords that we studied include a mixture of cell types. Recent studies in blood suggest that some of the interindividual variation in methylation is accounted for by differences in cellular content (Lam et al. 2012; Liu et al. 2013). We were unable to separate the cell types in the GUSTO umbilical cords as the tissues were frozen at collection. Our study involved only a subset of the SNPs in the genome, which included the 1 million SNPs on the OmniExpress + Exome arrays. Although missing SNPs could have accounted for additional variance in the VMRs, the 1 million SNPs were distributed relatively evenly across the genome and represented the majority of haploblocks. Finally, the CpG sites assessed by InfiniumHumanMethylation450 BeadChip are biased toward gene bodies and flanking regions, and therefore we did not cover intergenic regions thoroughly.

Our analysis was intended to estimate the relative influences of genotypic, environmental, and G × E interactive effects on the neonatal methylome. The models should not be considered as fully determinative of specific outcomes. Indeed, many of the environmental factors considered here are interdependent. Parity and maternal age, for example, are obviously correlated. Moreover, potential sources of environmental influence, such as socioeconomic status, were not considered. Similarly, the associations shown in Figure 6 derive from models that did not control for potentially important covariates. In addition, due to the number of predictive factors tested, it is difficult to ascribe significance to any individual association; instead, we report the models that best explain the variance in methylation values. Nevertheless our data serve to underscore the importance of G × E interactions and suggest that models of epigenetic variation should consider such interactive influences.

## Conclusions

To our knowledge, our report is the first attempt to quantify the relative influence of genotype and environment, as well as their interaction on the human epigenome. This quantification is important as many reports compare DNA methylation to phenotype independently of genotype. Our results strongly suggest that genotype is an essential factor in these relationships. In particular, it is an important question to address in neonates because the influence of prenatal environment on future disease risk is intensely studied with respect to subsequent risk of illness. Our findings suggest that such studies should include an assessment of the degree to which environmental influences are moderated by genotype.

## Methods

Briefly, 244 umbilical cord samples from healthy babies who were part of the GUSTO birth cohort study (Soh et al. 2013) were selected. Subject characteristics can be found in Table 1. Genotyping was performed on the Illumina OmniExpress + Exome array and processed in a standard fashion. DNA methylation profiling was performed on the InfiniumHumanMethylation450 BeadChip. Data were processed as described previously (Pan et al. 2012). Sex chromosome data were removed. A temporal batch effect was observed and removed using empirical Bayes methodology (COMBAT) (Johnson et al. 2007). All probes identified as cross-hybridizing in either Chen et al. (2013) or Price et al. (2013) were removed from the data set; 301,468 probes remained. (For detailed sample and data acquisition, see Supplemental Material.)

### Identification of interindividual CpGs

VMRs were detected as previously described (Ong and Holbrook 2014). A candidate VMR was defined as at least two spatially contiguous probes within 1 kb of each other and with MAD values greater than the 95th percentile. We expanded candidate regions to contain more than two probes, as long as the distance between any two neighboring probes within the region was not larger than 1 kb. For each VMR, the CpG with the highest MAD score was utilized as the representative CpG. These 1423 CpGs were used as the data set for subsequent analyses. In addition, the 1500 CpGs with the highest MAD scores outside of VMRs were also captured for further analysis.

### MethQTL analysis

Linear regression was performed for each of the 1423 VMR-CpGs, against all heterologous SNPs identified on the genome-wide arrays. For the purposes of these regressions, the genotype was coded as 1, 2, or 3 and treated as continuous whereby the heterozygote represents an intermediate state between the two homozygotes. In the model analysis below, genotype is categorical. The regressions were adjusted for sex. For each of the 1423 VMR-CpGs, we selected the CpG–SNP pair with the lowest *P*-value for further analysis. The linear regression analysis was performed in R.

### Model analysis

Methylation (*Meth*) at each CpG was subjected to the following models [(1), (2), (3)], and models were compared using Akiake information coefficients (AIC) (Akaike 1973).
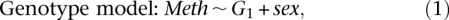
where *G*_1_ is the best SNP from previous MethQTL analysis, treated as a categorical variable.
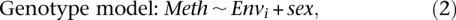
where *Env*_*i*_ is the phenotype (*i* = 1–19) (for its corresponding phenotype, see Table 1), which gave the lowest model AIC.
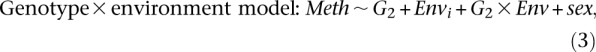
where *Env*_*i*_ is the phenotype (*i* = 1–19) (for its corresponding phenotype, see Table 1), which gave the lowest model AIC, and 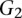 is the SNP on the same chromosome as *Meth*, which gave the lowest model AIC (also treated as a categorical variable).

The model with the lowest AIC was declared the “winner,” i.e., the model that best explained the Meth compared with the alternative models.

Akiake deltas were calculated as the difference between the AIC for the best model and the AIC for the next best model (Burnham and Anderson 2004).

Adjusted R^2^ was calculated by Equation 4:
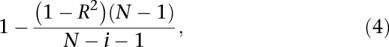
where 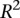 is sample R^2^; 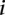, the number of predictors; and 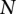, the total sample size.

### Segregated analysis

For each of the CpGs for which a G × E model had the lowest AIC, the subjects were segregated by the genotype, and regression against phenotype was performed in each genotypic group. Bonferroni correction was performed for 2963 regressions that were run.

### Genomic feature enrichment analysis

CpG island shores were defined as up to 2-kb regions from the CpG island start or end as per convention (Irizarry et al. 2009). CpG island shelves were next defined as another 2 kb from the shore boundaries, as specified in the GenomeStudio Methylation Module v1.8 User Guide from Illumina (Table 10) (http://supportres.illumina.com/documents/myillumina/90666eaa-0c66-48b4-8199-3be99b2b3ef9/genomestudio_methylation_v1.8_user_guide_11319130_b.pdf). Open seas are regions that are not islands, shores, or shelves. The TSS SwitchGear track in the UCSC Genome Browser was used to delineate human promoters, and genomic coordinates of human enhancers were obtained from the VISTA enhancers track (Visel et al. 2007). For individual VMR lists, we determined the total number of probes belonging to each of the six genomic categories (CpG island, south shore, north shore, south shelf, north shelf, and open sea) and also for a background list of 55,003 regions that are possible on the InfiniumHumanMethylation450K BeadChip. The *P*-value for enrichment of the region lists with each genomic category is computed by a hypergeometric test (one-tailed).

## Data access

The data from this study have been submitted to the NCBI Gene Expression Omnibus (GEO; http://www.ncbi.nlm.nih.gov/geo/) under accession numbers GSE53816 and GSE54445.
